# Experiences of pregnant women with genome-wide non-invasive prenatal testing in a national screening program

**DOI:** 10.1038/s41431-022-01248-x

**Published:** 2022-12-09

**Authors:** Karuna R. M. van der Meij, Qiu Ying. F. van de Pol, Mireille N. Bekker, Linda Martin, Janneke Gitsels-van der Wal, Elsbeth H. van Vliet-Lachotzki, Janneke M. Weiss, Robert-Jan H. Galjaard, Erik A. Sistermans, Merryn V. E. Macville, Lidewij Henneman, the Dutch NIPT Consortium, Karuna R. M. van der Meij, Elsbeth H. van Vliet-Lachotzki

**Affiliations:** 1 Department of Human Genetics, Amsterdam UMC, location Vrije Universiteit Amsterdam, Amsterdam, the Netherlands; 2 Amsterdam Reproduction and Development research institute, Amsterdam UMC, Amsterdam, the Netherlands; 3 Department of Obstetrics and Gynaecology, Utrecht University Medical Center, Utrecht, the Netherlands; 4 Department of Midwifery Science, AVAG, Amsterdam UMC, location Vrije Universiteit Amsterdam, Amsterdam, the Netherlands; 5 Amsterdam Public Health research institute Amsterdam UMC, Amsterdam, the Netherlands; 6 VSOP - Patient Alliance for Rare and Genetic Diseases, Soest, the Netherlands; 7 Department of Genetics, Radboud University Medical Center, Nijmegen, the Netherlands; 8 Department of Clinical Genetics, Erasmus MC, University Medical Center Rotterdam, Rotterdam, the Netherlands; 9 Department of Clinical Genetics, GROW School for Oncology and Developmental Biology, Maastricht University Medical Center, Maastricht, the Netherlands

**Keywords:** Ethics, Medical ethics

## Abstract

Pregnant women’s perspectives should be included in the dialogue surrounding the expanding offers of non-invasive prenatal testing (NIPT), especially now that technological possibilities are rapidly increasing. This study evaluated women’s experiences with the offer of genome-wide (GW) first-tier NIPT in a national screening program. A nationwide pre-and post-test questionnaire was completed by 473 pregnant women choosing between targeted NIPT (trisomies 21, 18 and 13 only) and GW-NIPT (also other findings) within the Dutch TRIDENT-2 study. Measures included satisfaction, reasons for or against choosing GW-NIPT, anxiety, and opinion on the future scope of NIPT. Most respondents (90.4%) were glad to have been offered the choice between GW-NIPT and targeted NIPT; 76.5% chose GW-NIPT. Main reasons to choose GW-NIPT were ‘wanting as much information as possible regarding the child’s health’ (38.6%) and ‘to be prepared for everything’ (23.8%). Main reasons to choose targeted NIPT were ‘avoiding uncertain results/outcomes’ (33.7%) and ‘not wanting to unnecessarily worry’ (32.6%). Nearly all respondents received a low-risk NIPT result (98.7%). No differences were found in anxiety between women choosing GW-NIPT and targeted NIPT. Most respondents were favorable toward future prenatal screening for a range of conditions, including life-threatening disorders, mental disabilities, disorders treatable in pregnancy and severe physical disabilities, regardless of their choice for GW-NIPT or targeted NIPT. In conclusion, women who chose first-tier NIPT were satisfied with the choice between GW-NIPT and targeted NIPT, and most women were favorable toward a broader future screening offer. Our results contribute to the debate concerning the expansion of NIPT.

## Introduction

In 2011, non-invasive prenatal testing (NIPT) was introduced into clinical practice [1]. NIPT uses cell-free DNA in maternal plasma derived from the placenta to screen for fetal aneuploidies. The advantages of NIPT (e.g., high sensitivity and low false-positive rate) compared to conventional screening methods resulted in a quick global dissemination [2]. NIPT implementation policies vary greatly between countries, from a first-tier screening test for the general population (private or within a publicly funded program), to a second-tier (contingent) test for women who have an increased risk for fetal aneuploidies [2, 3].

Though many NIPT technologies are based on whole-genome sequencing, NIPT is still primarily used for the detection of trisomy 21, 18, and 13 [4]. Genome-wide (GW) NIPT methods allow the reporting of additional findings such as rare autosomal trisomies, structural aberrations [5, 6] and sex chromosomal disorders [7]. Offering GW-NIPT to pregnant women has been the subject of scientific debate [8–10], since evidence regarding its clinical validity and utility (e.g., risks and benefits resulting from its use) is scarce [11]. GW-NIPT is not yet recommended by some professional societies [12, 13].

In commercial settings, many variations of NIPT panels are offered to screen for specific conditions other than the common trisomies, mainly microdeletions, e.g., DiGeorge syndrome [3]. However, these offers have been criticized for being marketed as highly accurate, while often giving false-positive results [14]. Due to declining sequencing costs and improving technology, the possibilities of NIPT are rapidly expanding. In the future, it will likely be possible to expand the scope of NIPT to include screening for fetal monogenic disorders [15], fetal-maternal risk-factors including preeclampsia, preterm birth and viral infections [16–18]. As the scope of NIPT widens, the complexity of the offer and women’s decision-making process increases.

In the Netherlands, NIPT is offered as a first-tier test within the national screening program as part of the TRIDENT-2 study. Women who elect to have NIPT can choose between a targeted (only the common trisomies) or genome-wide (GW) approach (also including chromosomal aberrations other than the common trisomies, except the sex chromosomes). A majority of pregnant women (78%) in the Netherlands chose GW-NIPT [5].

Counselling and informed decision making involving GW-NIPT is considered challenging, especially since findings can be clinically unclear [11]. Little is known regarding the psychosocial impact of the offer of GW-NIPT on women [19], such as increased feelings of anxiety. This study evaluates the experiences of women who are offered a first-tier GW-NIPT in a national screening program in order to weigh the benefits and potential harms, and ensure responsible implementation.

## Materials and methods

A pre- and post-test survey study using questionnaires was performed as part of the national TRIDENT-2 (TRIials by Dutch laboratories for the Evaluation of Non-invasive prenatal Testing) study. Approval for this survey study was granted by the VU University Medical Center Ethical committee (VUMC No. 2017.165).

### Setting

All pregnant women in the Netherlands are offered counselling regarding prenatal screening for fetal aneuploidy by a certified prenatal counsellor, mostly a primary care midwife. Since 2017, NIPT has been offered to all pregnant women as part of the TRIDENT-2 study. More information about the study protocol and the inclusion criteria have been published previously [5]. At the time of this survey study, after receiving pre-test counselling, participants could choose whether they wished to have no aneuploidy screening, first-trimester combined testing (FCT; no longer offered as of October first 2021) or NIPT (both FCT and NIPT were offered against comparable costs, 168 and 175 euros in 2018, respectively). Women who chose NIPT were also offered a choice between targeted NIPT (analyses of trisomies 21, 18 and 13 only) or GW-NIPT (reporting of other chromosomal findings with a size resolution of 10–20 Mb) at no additional cost [20]. Sex chromosomes are not analyzed. During counselling, parents were told that additional findings from GW-NIPT are all chromosome aberrations other than trisomies 21, 18 and 13. Additional findings can be present in the fetus, the placenta and very rarely in the mother, and follow-up testing is needed to confirm this. Additional findings can vary in seriousness from very severe to less severe. Approximately 4 in 1000 women who choose for GW-NIPT will receive a high-risk result for an additional finding [5]. No information was given on the positive predictive value for the different additional findings. In addition to the counselling, women were offered an information leaflet about prenatal screening and invited to visit an informational website. Women were informed by their prenatal counselor (obstetric care provider) prior to having NIPT how they will receive their result. A low-risk NIPT result is reported by the prenatal counselor, generally by phone. In case of an additional finding either the prenatal counsellor or clinical geneticist will contact the couple by phone. In case of an additional finding women are always referred to a clinical geneticist.

### Procedure and participants

Between October 2017 and November 2018, pregnant women from 28 midwifery practices and five hospitals in the Netherlands were invited to participate in this questionnaire study by their prenatal counsellor, and given a package containing an information letter and two questionnaires. The first questionnaire (Q1) was filled out by participants immediately after receiving pre-test counselling for prenatal screening regardless of their choice for screening. Results from this questionnaire have been previously published [21]. The second questionnaire (Q2) was filled out as soon as possible after receiving the NIPT result. Q1 was only available as a written questionnaire, while Q2 could be filled out either written or online, depending on women’s preference. The questionnaires were designed by a multidisciplinary group of stakeholders including a clinical geneticist, a midwife, a gynecologist, a psychologist, a patient representative, and a health scientist. The measures that we used to answer research questions are a combination of validated measures and measures created specifically for this study. Only women who chose NIPT and filled out and returned both questionnaires were included in this study.

### Measures

Respondents were asked to indicate their choice for NIPT analyses: GW-NIPT, targeted NIPT or do not recall. Reasons for or against choosing GW-NIPT were measured as an open-ended question in Q2. Women could explain their reasons for choosing either for or against GW-NIPT (‘Yes, I wanted GW-NIPT because’ or ‘No, I did not want GW-NIPT because’) in an open text box. Patients could provide multiple reasons.

State anxiety was measured using the six-item short-form of the Spielberger State-Trait Anxiety Inventory (STAI) both in Q1 and Q2 to compare anxiety levels pre- and post-test. Scores for each item, ranging from 1 (‘not at all’) to 4 (‘very much’), were combined and the total score was multiplied by 20/6 (range 20–80) [22, 23]. The anxiety score was dichotomized into normal (<43) and high (≥43) anxiety [24]. Cronbach’s alpha was 0.84, indicating good internal consistency. The extent to which participants were concerned regarding ‘Worries about bearing a physically or mentally handicapped child’ of the Pregnancy-Related Anxiety Questionnaire-Revised (PRAQ-R) were measured both in Q1 and Q2 by a 4-item subscale [25]. Scores ranged from 1 (‘absolutely not relevant’) to 5 (‘very relevant’) and were combined into a total score (range 4–20). Cut-off scores for dichotomizing PRAQ-R are not available, therefore the 90th percentile was chosen (cut-off score of ≥12) to signify high pregnancy-related anxiety [26]. Cronbach’s alpha was 0.86 indicating good internal consistency. STAI and PRAQ-R scores were analyzed separately for respondents who received a low-risk NIPT result or high-risk NIPT result. Respondents who filled out Q1 and Q2 on the same day (*n* = 16), or who filled out Q2 after giving birth (*n* = 2), were excluded from the STAI and PRAQ-R analysis.

Satisfaction was measured in Q2 by asking if participants were glad to have been offered NIPT, and if, in retrospect, they would have rather chosen a different test (GW-NIPT, targeted NIPT or FCT) or no test. Furthermore, women were asked if they were glad that NIPT could be used for the detection of additional findings, and if they would have rather not have wanted the option to choose between targeted and GW-NIPT (disagree/agree). At Q1, women were asked how they experienced making the decision for either targeted NIPT or GW-NIPT (easy/difficult) on a Likert scale.

Opinion on the future scope of prenatal screening was assessed using seven categories of disorders in Q2, based on a previous study among pregnant women before the introduction of NIPT [27]. Women were asked to indicate for each category (life-threatening, mental disability, physical disability, treatable disorders, fetal-maternal risk factors, late-onset disorders, or all disorders a woman wants) if they could choose, which of these types of disorders a prenatal test should screen for on a three-point scale (‘disagree’, ‘neither agree nor disagree’, ‘agree’).

Socio-demographic data were collected in Q1: age, education level, country of origin, religion, parity, health literacy, gestational age and informed choice [21].. Religion was measured by the question: ‘which denomination or ideology do you consider yourself to be?’ Answers were dichotomized: having no religious affiliation if answered ‘none’ or having a religious affiliation if an affiliation was selected. Informed choice was defined as a choice made with sufficient knowledge (≥5/7 questions correctly answered), value-consistent and adequately deliberated [21]. The knowledge questions focused on knowledge regarding prenatal screening, NIPT, FCT, invasive testing, and the meaning of possible test results [28]. A cut-off of 5/7 correctly answered questions was chosen to signify good knowledge according to Van den Berg et al. (2006), which is the guess corrected mid-point [29]. Deliberation was assessed using the Deliberation Scale [29]. This scale assesses evaluating the alternatives, thinking about the consequences and weighing up the pros and cons of prenatal screening.

### Statistical analysis

Descriptive analyses were used to describe participant characteristics and *t*-tests were used to test differences between groups. Wilcoxon paired ranks test was used to compare the difference in STAI and subscale-PRAQ-R results pre- and post-NIPT. Mann–Whitney U tests were used to compare differences in anxiety between women choosing GW-NIPT or targeted NIPT. Logistic regression analysis was performed to test for predictors for choosing GW-NIPT compared to women choosing targeted NIPT. Inductive content analysis was used to analyze and categorize reasons for or against choosing GW-NIPT, this was done by one researcher (KM) and discussed with another researcher (LH). Statistical significance was set at *p* < 0.05. All analyses were performed using IBM SPSS Statistics 26.

## Results

### Respondents

A total of 1561 pregnant women agreed to participate in the survey study and received both questionnaires (Q1 and Q2). Of these, 48.2% (*n* = 752) completed Q1, and 619 (82.3%) participants who completed Q1 also completed Q2 in an average of 8.4 days (SD 12.7) after receiving their test result. Women who chose FCT (*n* = 10) and women who chose not to have fetal aneuploidy screening (*n* = 135) were excluded from this study. The final sample consisted of *n* = 474 pregnant women who completed both questionnaires and had NIPT. At Q1, respondents had a mean age of 32.2 years (SD 3.9), were more often highly educated (71.2%) and of Dutch descent (86.9%) compared to the general Dutch obstetric population (Table 1). At Q2, women had a mean gestational age of 14.8 weeks (SD 2.7), compared to 10.9 weeks (SD 1.7) at Q1. Six respondents (1.3%) received a high-risk NIPT result: three for trisomy 21, one for trisomy 18, and two for a structural aberration (additional finding) as a result of GW-NIPT. In total, 78% of the respondents made an informed choice for NIPT.

**Table 1:** Respondent characteristics *n* = 474, compared to the general obstetric population.

	*N* (%)	General Dutch obstetric population (%)
Age (years) (Q1) (missing 1)		
≤29	111 (23.5)	(33.5)^a^
30–34	238 (50.3)	(40.8)^a^
≥35	124 (26.2)	(25.7)^a^
Education level (missing 1)^b^		
Low	16 (3.4)	(12.3)^a^
Intermediate	120 (25.3)	(35.4)^a^
High	337 (71.2)	(50.9)^a^
Country of origin (missing 2)^c^		
Dutch	410 (86.9)	(67.0)^a^
Other-Western	39 (8.3)	(11.4)^a^
Non-Western	23 (4.9)	(21.6)^a^
Religious affiliation (missing 6)		
Non-religious	327 (69.9)	~(63)^a^
Religious	141 (30.1)	~(37)^a^
Parity (missing 2)		
Primiparous	234 (49.5)	(45.4)^a^
Multiparous	239 (50.5)	(55.6)^a^
Health literacy (missing 2)^d^		
Adequate	414 (87.7)	
Inadequate	58 (12.3)	
Gestational age (Q1) (weeks) (missing 2)		
≤10	162 (34.3)	
11–14	299 (63.3)	
≥15	11 (2.3)	
Informed choice for having NIPT (missing 103)^e^		
Informed choice	288 (77.6)	
Uninformed choice	83 (22.4)	
Choice for NIPT analysis (Q2) (missing 1)		
Genome-wide NIPT	362 (76.5)	(77.6)^f^
Targeted NIPT	103 (21.8)	(23.4)^f^
Do not recall	8 (1.7)	
NIPT result (Q2) (missing 3)		
Low-risk	465 (98.7)	(97.7)^f^
High-risk: trisomies 21,18,13	4 (0.9)	(0.46)^f^
High-risk: finding other than trisomies 21,18, or 13	2 (0.4)	(0.36)^f^

*NIPT* non-invasive prenatal test, *Q* questionnaire, pre- (Q1) or post-test (Q2).

^a^Source: Statistics Netherlands. Maternal age, country of origin and parity of all livebirths in the Netherlands in 2019. Education level of women in the Netherlands aged 25–45 years in 2019. Religious affiliation of women in the Netherlands aged 25–35 years in 2018.

^b^Education levels were categorized as low: elementary school, low level secondary school or lower vocational training; intermediate: high level secondary school or intermediate vocational training; or high: high vocational training or university.

^c^Country of origin was categorized as Dutch (according to Statistics Netherlands) if both parents were born in the Netherlands; other Western: one or both parents were born in Europe (excluding Turkey), North America, Oceania, Indonesia or Japan; non-Western: one or both parents were born in Africa, Latin-America, Asia (excluding Indonesia or Japan) or Turkey. Maternal country of birth was leading if both parents were born abroad.

^d^Health literacy was measured using the three-item set of brief screening questions [24]. Categorized as inadequate if answered anything other than ‘never’ or ‘occasionally’ on one or more questions.

^e^Based on van der Meij et al. 2022 [21]. Women with a neutral attitude (*n* = 103) were excluded from the calculation of informed choice.

^f^Based on van der Meij et al. 2019 [5].

### Choosing for or against GW-NIPT

Most of the survey respondents elected to have GW-NIPT (362/473; 76.5%), 21.8% chose targeted NIPT (103/473), and 1.7% could not recall the decision (8/473). Univariate logistic regression analysis revealed that the variables age, education level, country of origin, religion, parity, health literacy and gestational age at Q1 were all not significantly associated with the decision for either GW or targeted NIPT. We did not find differences in the levels of informed choice between participants choosing GW-NIPT or targeted NIPT (*p* = 0.498).

A total of 336/362 women gave 376 reasons for choosing GW-NIPT over targeted NIPT (Table 2); 26 women did not specify their decision. The main reasons to choose GW-NIPT were: ‘wanting as much information as possible regarding the child’s health’ (*n* = 130, 38.6%), ‘to be prepared for everything’ (*n* = 80, 23.8%) and ‘making optimal use of the test’s abilities’ (e.g., no additional costs and the analysis is being done anyway) (*n* = 47, 13.9%). For 4.8%, receiving information about the woman’s own health was a reason to choose GW-NIPT. A total of 86/103 respondents who chose against GW-NIPT reported 108 reasons; 17 women did not specify their decision. The main reasons to choose against GW-NIPT were: ‘avoiding uncertain results/outcomes’ (*n* = 29, 33.7%) and ‘not wanting to unnecessarily worry’ (*n* = 28, 32.6%). Moreover, 11.6% did not think the results of GW-NIPT would be reliable.

**Table 2:** Reasons for choosing GW-NIPT (*n* = 336 women) and reasons against choosing GW-NIPT (*n* = 86 women).

Reasons for choosing GW-NIPT	Total number of reasons *n* = 376 given by 336 women	Reasons against choosing GW-NIPT	Total number of reasons *n* = 108 given by 86 women
	*N* (%)		*N* (%)
I want as much information as possible regarding the health of my child	130 (38.6)	To avoid uncertain results/outcomes	29 (33.7)
To be prepared for everything	80 (23.8)	I do not want to unnecessarily worry	28 (32.6)
I want to make optimal use of the test’s abilities	47 (13.9)	I do not want or need this information	11 (12.8)
I want to be reassured about the health of my child	24 (7.1)	The results are not reliable/reliability is unclear	10 (11.6)
To be able to make informed (reproductive) decisions about the current pregnancy	21 (6.3)	I only want to know about trisomies 21, 18, and 13	9 (10.5)
General interest	19 (5.7)	It is impossible to know everything	8 (9.3)
I want to receive information about my own (the woman’s) health	16 (4.8)	Other^b^	6 (7.0)
To gain certainty	12 (3.6)	I do not want to know about mild conditions	5 (5.8)
It feels like the right decision	11 (3.3)	I would not terminate in case of additional findings	2 (2.3)
Because of (known or unknown) family history of disease or risk factors	8 (2.4)		
Other^a^	8 (2.4)		

^a^Other reasons included ‘my partner wanted it’ and ‘it seemed important’.

^b^Other reasons included ‘I do not want to know about my own health’ and ‘I only did the test because of my age’.

### Anxiety

Pre-test, respondents scored a mean state anxiety level of 32.7 (SD 9.6) on the STAI, which declined significantly to 28.2 (SD 8.0) after receiving a low-risk NIPT result (*p* < 0.001). An elevated post-test STAI score (≥43) was found in 5.7% of the respondents. Respondents had a mean pre-test pregnancy-related anxiety (PRAQ-R) score of 9.2 (SD 3.1), which declined significantly to 8.2 (SD 2.9) after receiving their low-risk NIPT result (*p* < 0.001). An elevated post-test PRAQ-R (≥12) score was found in 14.1% of the respondents. No significant differences were found in both pre- and post-test anxiety levels (STAI and PRAQ-R) between women choosing for GW-NIPT vs. targeted NIPT.

Four out of the six participants in this study who received a high-risk NIPT result, filled out the second questionnaire after having done invasive diagnostic testing. One participant had not yet had invasive testing but wanted to have it, and one participant had additional blood tests performed. These six high-risk women had mean pre-test STAI and PRAQ-R scores of 35.6 (SD 12.4) and 8.5 (SD 2.9), respectively. After receiving the high-risk NIPT result, their anxiety levels increased to 57.3 (STAI; SD 22.0) and 14.8 (PRAQ-R; SD 6.3), respectively.

### Satisfaction

Of all respondents, 99.2% were glad to have been offered NIPT. Almost all respondents (99.6%) reported that, in retrospect, they would make the same choice. One woman reported that, in retrospect, she would have preferred not to have done any screening, and one woman reported that she would have preferred to have a targeted NIPT instead of GW-NIPT (no reason for this was given); both had received a low-risk result. Nearly all (98.9%) of the respondents who elected to have GW-NIPT were glad that NIPT could be used to detect findings other than trisomies 21,18 and 13, compared to 39.4% of respondents who chose targeted NIPT. Overall, 90.4% of respondents were glad that they could choose between targeted NIPT and GW-NIPT. Of the respondents choosing GW-NIPT, 3.3% agreed that they would rather not have had to option to choose between targeted NIPT and GW-NIPT, compared to 31.6% of respondents who chose targeted NIPT (*p* < 0.001). For respondents who chose GW-NIPT, 18.2% found choosing between targeted NIPT and GW-NIPT (somewhat) difficult, compared to 40.2% of respondents who chose targeted NIPT (*p* < 0.001). After receiving their low-risk result, 95.4% of all women were reassured and 96.8% did not regret testing. After receiving a high-risk result, one woman (1/6; 16.7%) reported regretting that she chose NIPT.

### Future scope of prenatal screening

**Fig. 1:**
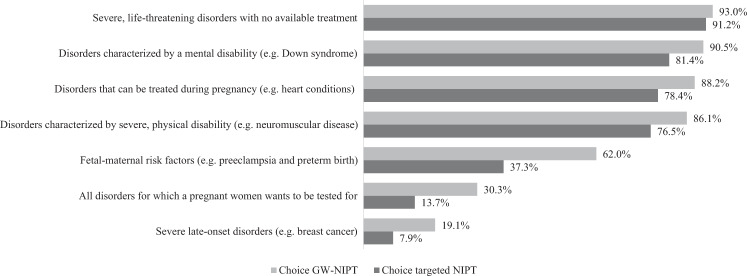
Opinion on the future scope of prenatal screening sorted by choice for GW-NIPT (*n* = 362) or targeted NIPT (*n* = 103). Bars represent percentage of agreement for each category from participants who chose GW-NIPT (light grey) or targeted NIPT (dark grey).

Figure 1 shows the percentage of respondents that agreed with the offer of a first-tier prenatal test aimed at screening for several types of conditions, stratified by their choice for GW-NIPT or targeted NIPT. The majority of respondents from both groups agreed with screening for: severe untreatable life-threatening disorders (93.0–91.2%), disorders characterized by a mental disability (90.5–81.4%), disorders that can be treated during pregnancy (88.2–78.4%) and severe physical disabilities (86.1–76.5%). Compared to women who chose GW-NIPT, women who opted for targeted NIPT less often agreed with screening aimed at fetal-maternal risk factors (62.0% vs. 37.3%), all disorders a woman wants to be tested for (30.3% vs. 13.7%) and severe late-onset disorders (19.1% vs. 7.9%).

## Discussion

This study reports the experiences of pregnant women who were offered first-tier GW-NIPT in a national screening program. Most women were satisfied with the choice between GW-NIPT and targeted NIPT, though women choosing targeted NIPT were less satisfied with this option. The main reasons to choose GW-NIPT were ‘wanting as much information as possible’ and ‘to be prepared for everything’. The main reasons to choose targeted NIPT were ‘to avoid uncertain results’ and ‘not wanting to unnecessarily worry’. No differences were found in anxiety levels between women choosing GW-NIPT or targeted NIPT. The majority of women agreed with a (hypothetical) future offer of a prenatal test aimed at screening for severe untreatable life-threatening disorders, disorders characterized by a mental disability, disorders that can be treated during pregnancy and severe physical disabilities.

The main reasons for participants to choose GW-NIPT were ‘wanting as much information as possible about the health of the child’ and ‘to be prepared for everything.’ Research has shown that, when given the choice, pregnant women prefer to receive more information about the health status of their child [30]. In a study comparing the views of pregnant women and healthcare professionals from nine different countries, pregnant women were willing to accept a less accurate test to obtain more information on fetal chromosomal status, compared to healthcare professionals who placed a greater importance on test accuracy [31]. In our study, most women were glad that they were given the choice between GW-NIPT or targeted NIPT, though percentages of agreement were lower in the targeted group. It has been suggested that enabling parents to determine which type of prenatal test they prefer is good for their wellbeing and post-test satisfaction [32], although high-quality pre-test counselling is required to achieve informed choice. Moreover, professionals may find it too burdensome to provide a woman with different choices [33]. Finally, it should be noted that requiring an out-of-pocket payment for GW-NIPT at the same price as targeted NIPT might inadvertently steer women to choose GW-NIPT to ‘get their money’s worth.’ It would be valuable to study whether pregnant women’s decision-making for GW-NIPT might change if the test was free of charge.

For 4.8% of women, receiving information about their own health was (one of) the main reason(s) to choose GW-NIPT. While it is possible to detect constitutional or acquired maternal chromosome aberrations such as cancer [11, 34], this is not the purpose of NIPT, nor is NIPT suitable for this type of screening [20]. The main reasons to choose for targeted NIPT were ‘avoiding uncertain results/outcomes’ and ‘not wanting to unnecessarily worry.’ These reasons may reflect women’s perceptions of the current lack of knowledge regarding the clinical validity and utility of GW-NIPT, suggesting that women who choose against GW-NIPT do so to avoid uncertainty and worries, and not because they do not want to know about other fetal disorders.

In our study, most women were satisfied with having NIPT, which may not be surprising as most women received a low-risk result. Pregnant women may, however, not always be adequately prepared for their NIPT result or be aware of its limitations. A US study of pregnant women who received an inconclusive, false-positive or false-negative NIPT result showed that many reported feeling misled by the information they received, and the authors concluded this was due to inadequate pre-test counseling [35]. This highlights the importance of high-quality pre-test counselling.

Similar to other studies, we found that anxiety levels decreased significantly after receiving a normal aneuploidy screening result [36]. We did not find differences in pre- or post-test anxiety levels between women choosing GW-NIPT compared to women choosing targeted NIPT. Six women in our study received a high-risk result. In line with literature, anxiety levels increased after receiving a high-risk result in prenatal screening [19, 36]. Of the low-risk participants in our study 14.1% had an elevated post-test PRAQ-R score. Tough anxiety during pregnancy is common [37], interventions that address anxiety among pregnant women are scarce [38]. This may highlight a need for interventions that address mental health in pregnancy. A systematic review on this topic showed that women valued individual or group discussions about their anxiety [39].

The majority of our respondents were favorable toward widening the scope of the prenatal screening regardless of their choice for GW-NIPT or targeted NIPT, though percentages of agreement were higher in the GW-NIPT group. Overall, our results are comparable to a Dutch study among pregnant women performed before the introduction of first-tier NIPT [27]. A qualitative study among pregnant women from Quebec and Lebanon on the scope of GW-NIPT found that the severity of the condition, the time of onset, and the perceived quality of life of the child were important factors when considering the acceptability of GW-NIPT [40]. Similar to our results, most respondents were favorable toward screening for severe early-onset (e.g., onset shortly after birth or in childhood) and treatable conditions [40]. An Australian questionnaire study also found that women were interested in screening for medical conditions with an early-onset [41]. In our study, screening for fetal-maternal risk-factors was acceptable for 62% of women choosing GW-NIPT and 37% of women choosing targeted NIPT. When screening for fetal-maternal risk-factors, the aim of screening shifts from enabling reproductive autonomy to prevention of mortality and morbidity [42]. When these two different types of screening are offered simultaneously, there is a risk that parents become confused about the purpose of testing, and which values should be considered, making counselling and informed decision-making more challenging [42]. Some researchers have proposed to separate screening with different aims and not offer dual-purpose screening [42]. Similar to previous studies, only a small minority of our respondents supported testing for severe late-onset disorders with NIPT [27, 41]. As these disorders are not expected to lead to pregnancy termination, the interests and ‘right to an open future’ of the child should be considered [42]. This will require balancing the parents’ wish to be as informed as possible against the risk of exposing the future child to harmful information.

An objection against widening the scope of NIPT is that the information provision about many different types of disorders may challenge professionals’ counselling and cause information overload, hindering parents’ informed decision making [33]. This may undermine rather than enhance reproductive autonomy [42]. Counselling models have been proposed for prenatal screening that focus on creating a dialogue about overarching information and personal values as opposed to providing couples with value-free technical information, mitigating the issue of information overload [43]. However, research is needed to determine whether these models work in practice.

### Strengths and limitations

This is one the first studies to describe women’s experiences with the offer of first-tier GW-NIPT. The majority of women (76.5%) in our study population chose to have GW-NIPT, which is similar to the actual percentage of women choosing for GW-NIPT in the Netherlands in 2017 (78%) [5]. Our study sample primarily consisted of highly educated Dutch women and the questionnaires were only available in Dutch, limiting the generalizability of our results. This study mainly consisted of women who received a low-risk NIPT result, which may have affected the outcome of the study. Within the TRIDENT-2 study research is being conducted to investigate the psychological impact of receiving a high-risk GW-NIPT additional finding. Another limitation arises regarding the future scope of NIPT: respondents may have had different levels of awareness and knowledge of the categories of conditions described in the questionnaire. Examples were provided for five of the seven categories, which could have affected the responses. Qualitative research can be used for a more in-depth exploration of women’s perspectives on the scope of NIPT.

## Conclusion

Most women having NIPT were glad to have been offered the choice between GW-NIPT and targeted NIPT, though agreement was lower for the targeted group. The majority of women were favorable toward widening the future scope of prenatal screening regardless of their choice for GW-NIPT or targeted NIPT. The results of this study can inform the dialogue surrounding the expansion of NIPT, can contribute to the development of governmental and professional guidelines for GW-NIPT and inform the information provided to pregnant women.

## Supplementary information

The Dutch NIPT Consortium

## Data Availability

The datasets generated during the current study are available from the corresponding author on reasonable request.
