# Supplementary material for: Experiences of pregnant women with genome-wide non-invasive prenatal testing in a national screening program
Source: Eur J Hum Genet. 2022 Dec 9;31(5):555–61. doi: 10.1038/s41431-022-01248-x (PMC10172316; doi:10.1038/s41431-022-01248-x)
Supplement: Supplementary file 1 — The Dutch NIPT Consortium [file 41431_2022_1248_MOESM1_ESM.docx]

**The Dutch NIPT Consortium**

*The Dutch NIPT Consortium consists of obstetric care givers, laboratory specialists and other professionals from:*

**Amsterdam UMC***

Human Genetics:

Dr. E.A. Sistermans (also for the Dutch Association of Clinical Genetic Diagnostic Laboratories (VKGL))

Prof. L. Henneman

Dr. A. Polstra

E. Voorhoeve MSc

S.L. Zelderen-Bhola MSc

Dr. E.M.J. Boon

Dr. M.P.R. Lombardi

I.M.C. Bakker MSc

E.J. Bradley BSc

C. Louwerens-Zintel BSc

M. Smit BSc

Dr. M.C. van Maarle

M.B. Tan-Sindhunata MSc

K. van der Meij MSc

Prof. H. Meij

Obstetrics and Gynecology:

Dr. C.J. Bax (also for the Dutch Organization of Obstetrics and Gynecology (NVOG))

Prof. E. Pajkrt

Dr. I.H. Linskens

Midwifery Science AVAG:

Dr. L. Martin

Dr. J.T. Gitsels-van der Wal

**Erasmus Medical Center, Rotterdam***

Clinical Genetics:

Dr. R.J.H. Galjaard (also for the Dutch Association of Clinical Geneticists (VKGN))

Dr. D. Van Opstal

Dr. M.I. Srebniak

Dr. F.M. Sarquis Jehee

I.H.I.M Hollink MSc

Dr. F. Sleutels

W. de Valk BSc

W.H. Deelen BSc

Dr. A.M.S. Joosten

Dr. K.E.M. Diderich

M.E. Redeker

Obstetrics and Gynecology:

Dr. A.T.J.I Go

Dr. M.F.C.M. Knapen

Dr. S. Galjaard

Dr. A.K.E. Prinsen

Information & Technology:

A.P.G. Braat

**Leiden University Medical Center**

Clinical Genetics:

Dr. M.J.V. Hoffer

Dr. N.S. den Hollander

Obstetrics and Gynecology:

Dr. E.J.T. Verweij

Dr. M.C. Haak

****NIPT laboratories***

**Maastricht University Medical Center***

Clinical Genetics:

Dr. M.V.E. Macville

Dr. S.J.C. Stevens

Dr. A. van der Wijngaard

L.H. Houben BSc

M.A.A. van Esch-Lennarts BSc

L. Hamers BSc

A.G.P. Jetten, BSc

Dr. S.A.I. Ghesquiere

Dr. B. de Koning

Dr. M. Zamani Esteki

C.J. Heesterbeek MD

Prof. C.E.M. de Die-Smulders

Prof. H. Brunner

Obstetrics and Gynecology:

M.J. Pieters MD (also for the Regional Centers Prenatal Screening in The Netherlands)

Dr. A.B.C. Coumans

**Radboud University Medical Center, Nijmegen**

Clinical Genetics:

Dr. D.F.C.M. Smeets

Dr. B.H.W. Faas

Dr. D. Westra

Dr. M.M. Weiss

I. Derks-Prinsen BSc

Dr. I. Feenstra

Dr. M. van Rij

Obstetrics and Gynecology:

Dr. E. Sikkel

**University Medical Center Groningen**

Clinical Genetics:

Dr. R.F. Suijkerbuijk

Dr. B. Sikkema-Raddatz

Prof. I.M. van Langen

Dr. K. Bouman

Obstetrics and Gynecology:

L.K. Duin MD

**University Medical Center Utrecht**

Clinical Genetics:

Dr. G.H. Schuring-Blom

Dr. K.D. Lichtenbelt

Obstetrics and Gynecology:

Prof. M.N. Bekker

**The Royal Dutch Organization of Midwives (KNOV)**

Dr. A.J.E.M. van der Ven

**VSOP Patient Alliance for Rare and Genetic Diseases**

E. van Vliet-Lachotzki MD

**Erfocentrum, Dutch National Genetic Resource and Information Center**

J. Pot

**Dutch NIPT Consortium, project manager**

S. van ‘t Padje
